# Adsorption/Desorption Behaviors and SERS Chemical Enhancement of 6-Mercaptopurine on a Nanostructured Gold Surface: The Au_20_ Cluster Model

**DOI:** 10.3390/molecules26175422

**Published:** 2021-09-06

**Authors:** Nguyen Thi Nhat Hang, Nguyen Thanh Si, Minh Tho Nguyen, Pham Vu Nhat

**Affiliations:** 1 Faculty of Food Science and Technology, Thu Dau Mot University, Thu Dau Mot 590000, Vietnam; hangntn@tdmu.edu.vn; 2 Department of Chemistry, Can Tho University, Can Tho 900000, Vietnam; sidoublet276@gmail.com; 3 Institute for Computational Science and Technology (ICST), Ho Chi Minh City 700000, Vietnam

**Keywords:** 6-mercaptopurine, gold nanostructure, gold clusters, the Au_20_ cluster model, IR spectra, SERS chemical enhancement, DFT computations

## Abstract

Computational approaches are employed to elucidate the binding mechanism and the SERS phenomenon of 6-mercaptopurine (6MP) adsorbed on the tetrahedral Au_20_ cluster as a simple model for a nanostructured gold surface. Computations are carried out in both vacuum and aqueous environments using a continuum model. In the gaseous phase and neutral conditions, interaction of 6MP with the gold cluster is mostly dominated by a covalent Au−S bond and partially stabilized by the Au⋅⋅⋅H−N coupling. However, in acidic solution, the nonconventional Au⋅⋅⋅H−S hydrogen-bond becomes the most favorable binding mode. The 6MP affinity for gold clusters decreases in the order of vacuum > neutral solution > acidic medium. During the adsorption, the energy gap of Au_20_ substantially declines, leading to an increase in its electrical conductivity, which can be converted to an electrical noise. Moreover, such interaction is likely a reversible process and triggered by either the low pH in sick tissues or the presence of cysteine residues in protein matrices. While N−H bending and stretching vibrations play major roles in the SERS phenomenon of 6MP on gold surfaces in neutral solution, the strongest enhancement in acidic environment is mostly due to an Au⋅⋅⋅H−S coupling, rather than an aromatic ring-gold surface π overlap as previously proposed.

## 1. Introduction

**Scheme 1:**
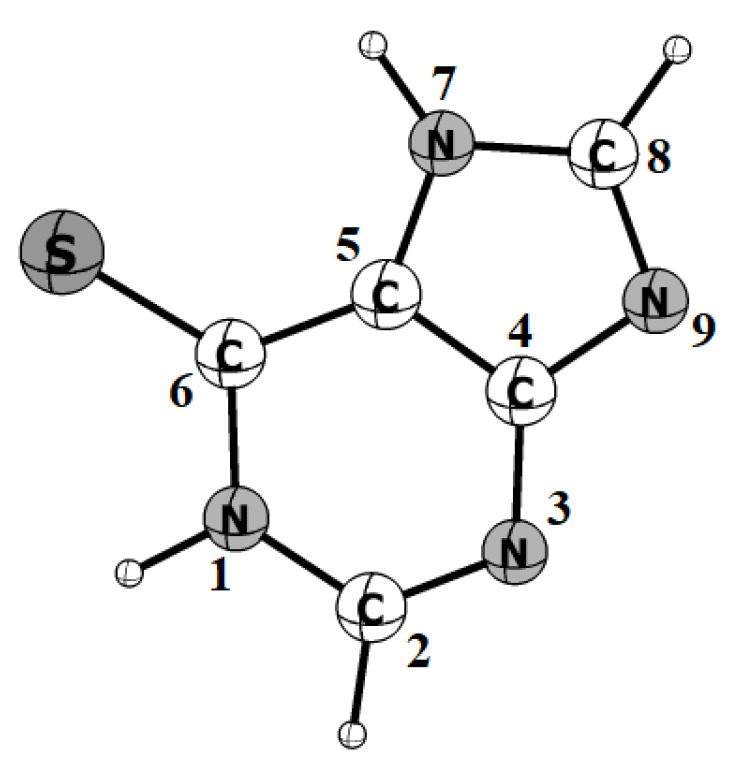
Chemical structure and atom numbering for 6-mercaptopurine (6MP).

Sulfur-containing compounds with a C=S functional group often exhibit a variety of biological activities and play vital roles in pharmaceutical applications. From a chemical structure viewpoint such a class of drugs can be categorized as either a thiourea S=C(NR_2_)_2_ or an artificial DNA nucleobase [1]. Behaving as a nucleobase analogue, the C=S double bond in 6-thiopurine or 6-mercaptopurine (6MP) acts as a surrogate for the natural C=O compounds (Scheme 1). Such a sulfur analogue of adenine is commonly used in the conventional chemotherapy treatment of patients with acute lymphoblastic leukemia, Crohn’s disease, and ulcerative colitis [1,2,3], for its prevention of purine nucleotide biosynthesis [1]. However, the adsorption of 6MP is rather poor, and its therapeutic intake may cause several adverse reactions such as diarrhea, nausea, vomiting, loss of appetite, hair loss, and inflammation of the mouth [4,5]. In this context, it is of great significance to find an appropriate carrier to deliver the drug where it is needed, with the aim of enhancing its therapeutic effects and consequently reducing the unwanted side effects. In addition, development of a simple but powerful method for selective detection of the drug is also of great pharmaceutical interest [6].

In recent decades, nanostructured materials have become more and more popular in biomedical fields in the design of drug delivery systems, diagnosis agents, imaging, and bio-sensors [7,8]. Among the various types of nano-carriers, i.e., dendrimers [9,10], polymers [11,12], liposomes [13,14,15] and metal nanoparticles [16,17,18,19,20], used in drug delivery processes, gold nano-particles (AuNPs) have received a great deal of attention owing to their unique physicochemical properties, high stability, low cytotoxicity, easy functionalization capability, and other relevant features [21,22,23,24,25]. Many valuable outcomes of the use of AuNPs as therapeutic agents, e.g., enhancing the therapeutic effect of the drug, allowing an efficient drug delivery, increasing the therapeutic retention time in the circulation, and improving the target specificity of treatments, have also been well documented in the current literature [26,27,28,29]. Taking advantage of such effects, numerous studies have been carried out to establish suitable techniques for detection of drugs and biomolecules in different media using gold particles [30,31,32,33,34,35]. In particular, the molecular specificity of Raman spectroscopy combined with metallic nano-particles to enhance the signal intensity, which is known as the surface enhanced Raman spectroscopy (SERS) technique, has been used for the study of not only simple molecules such as nucleobases and amino acids, but also for more complex functional structures, i.e., nucleic acids and proteins [36,37,38]. Owing to its efficiency and advantages such as low detection limits, greatly enhanced vibrational positions, and high selectivity for adsorbed molecules, the SERS method has been established as a conventional but highly promising analytical technique for detection of trace amounts of many organic contaminants [39,40].

A number of metals have been identified as capable of producing the SERS phenomenon, including silver, gold, copper, nickel, iron, ruthenium, etc. [41]. Coinage metals turn out to be more suitable overall for use as SERS substrates, since they typically exhibit a localized surface-plasmon resonance in the visible and near-infrared wavelength range where most Raman measurements occur [42]. However, because of several complicating factors including chemical enhancement effects, the Raman band intensity of adsorbates is quite differentiated among specific metal surfaces, and it is rather difficult to predict when a SERS phenomenon can properly be observed. Therefore, a deeper insight into the metal substrate and molecule binding mechanisms is of critical importance for a more comprehensive interpretation of SERS spectra. Several reports on the interactions of 6MP with nanostructured metal surfaces in terms of both experimental and theoretical aspects are available in the recent literature [43,44,45,46]. In order to elucidate the nature of these interactions, validate relevant thermodynamic parameters, as well as interpret or predict spectroscopic properties from the vibrational, electronic absorption spectra, density functional theory (DFT) approaches are commonly selected. Small metallic clusters are typically used as reactant models to investigate the binding mechanism of organic compounds on nanoparticle surfaces. Rashidpour et al. [47] analyzed the adsorption of 6MP on an Au_10_ surface and found that the conjugation is energetically preferred via the S and S-N sites of the drug molecule. Ren and co-workers [48] also investigated the structures, bonding features, and stability of the complexes of 6MP with an Ag_8_ cluster by means of DFT (B3LYP) calculations. In a recent study [49] we elucidated the binding mechanism of both 6-mercaptopurine and 6-thioguanine to the Au_6_ and Au_8_ clusters. Gold nanoclusters are found to exhibit appropriate characteristics for design of bio-sensing and targeted drug delivery of thione-containing compounds. Irrespective of previous efforts devoted to the study of 6MP binding to gold surfaces [46,49], there are many inherent limitations associated with these studies, and the SERS chemical enhancement of thione-containing drugs by nanostructured gold surfaces is still not fully understood. As a matter of fact, the cluster models used in our recent studies were not large enough to effectively represent the gold nanosurface. Therefore, we set out in the present study to perform DFT calculations to further examine the adsorption/desorption behaviors and the electronic response resulting from the 6MP molecule adsorbed on the larger, stable, and abundant Au_20_ cluster as a representative gold nanostructured surface. More specifically, the SERS spectrum of the drug adsorbed on the golden surface is generated to interpret available experimental data. A chemical enhancement mechanism is also proposed to provide deeper insights into the SERS phenomenon. As a result of the relative effects of gold, theoretical studies of its derivatives are challenging but important as unexpected phenomena and properties have often been obtained.

## 2. Computational Methods

Quantum chemical calculations were performed for both binding features and energetic/electronic properties using the Gaussian 16 program package [50]. Geometries of all systems studied were located with the Perdew–Burke–Ernzerhof (PBE) functional [51] in combination with a mixed basis set, i.e., the effective core potential (ECP) cc-pVDZ-PP [52] for the Au element and cc-pVTZ for all atoms in the 6MP molecule. The PBE functional combined with the cc-pVDZ-PP/cc-pVTZ basis sets has been shown to describe relatively well the interactions between gold atoms and sulfur-containing molecules [49,53]. Its use greatly facilitates a direct comparison with previous results. As both the Au_20_ cluster and all adsorbed species have a closed electron shell in their ground state, we considered only the singlet state. The effect of the solvent, an aqueous solution considered in this study, was simulated using the continuum model, namely, the integral equation formalism-polarizable continuum model (IEF-PCM) [54]. This model has been proved to properly describe both electrostatic and non-electrostatic interactions, and thus could provide us with a sufficient physical description of the solvent environment [55].

Harmonic vibrational frequencies were subsequently computed at the same level to validate the stationary points as equilibrium structures and to estimate the zero-point energy (ZPE) corrections. The Gibbs free energy of the interaction was then calculated with Equation (1):

ΔG° (298 K) = ΔE + ΔZPE + ΔTCG(1)

where ΔE is the relative electronic energy at 0 K, ΔZPE the relative zero-point energy at 0 K, and ΔTCG the relative changes in Gibbs free energy going from 0 to 298 K. The harmonic vibrational frequencies were also employed to simulate the Raman/SERS spectra (without scaling).

The strength of the interactions between both gold cluster and drug molecule was evaluated via the binding energy $E_{b}$. This parameter is simply defined as the total energy difference between those of the Au_20_∙6MP complexes and the isolated species by the following Equation (2):

(2) $$E_{b}=\left(E_{{\mathrm{Au}}_{20}}+E_{6\mathrm{MP}}\right)-E_{{\mathrm{Au}}_{20}\cdot 6\mathrm{MP}}$$

where $E_{{\mathrm{Au}}_{20}\cdot 6\mathrm{MP}}$ is the total energy of the Au_20_∙6MP complexes, whereas $E_{{\mathrm{Au}}_{20}}$ and $E_{\mathrm{MP}}$ are the energies of the Au_20_ nanocluster and 6MP molecule, respectively. Thus, a positive value of $E_{b}$ corresponds to an exothermic adsorption, and vice versa. A more positive $E_{b}$ indicates 6MP’s stronger affinity for metals.

Energies of frontier molecular orbitals, i.e., HOMO and LUMO, the HOMO–LUMO energy gap (E_g_), and the change of E_g_ upon binding of the drug to gold clusters were also computed to evaluate the effect of interacting species on each other. The E_g_ is a useful parameter for determining the kinetic reactivity of materials [56], and its change upon the adsorption process can be used to evaluate the sensitivity of an adsorbent to an adsorbate. Furthermore, the Fermi level energy (E_F_) and the work function (Φ) of the systems considered were computed by Equations (3) and (4):

(3) $$E_{F}=\frac{E_{\mathrm{HOMO}}+{\;E}_{\mathrm{LUMO}}}{2},$$

(4) $$\Phi =V_{e}\left(+\infty \right)-E_{F}$$

where $E_{\mathrm{HOMO}}$ and $E_{\mathrm{LUMO}}$ are HOMO and LUMO energies, respectively; $V_{e}\left(+\infty \right)$ is the electrostatic potential energy of an electron away from a structured surface.

Φ refers to the energy required for removing an electron from the Fermi level [57]. Thus, we have $\Phi =-E_{F}$ for $V_{e}\left(+\infty \right)=0$. Let us mention that Equation (3) is only valid for non-degenerate frontier MOs.

In this study, the impact of 6MP on the $E_{F}$ and Φ values of Au_20_ was investigated as the main factor in Φ-type sensors using a Kelvin oscillator to quantify Φ during the interaction [58]. Potential changes in the Φ of a sensor arising from the adsorption disturb the gate voltage and generate an electrical noise, which is a suitable indicator for recognizing the presence and attachment of a chemical [59].

## 3. Results and Discussion

### 3.1. Structures and Energetics

**Figure 1:**
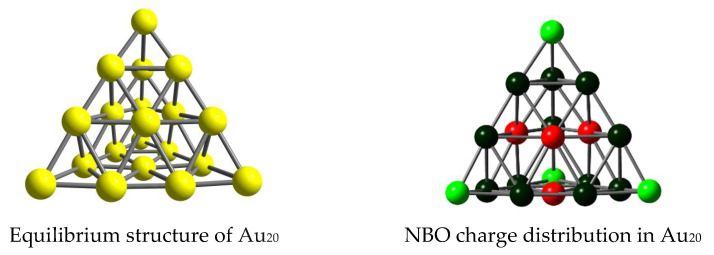
Geometry and NBO charge distribution in the tetrahedral Au_20_ cluster. Au color range.: green, more positive than 0.06 au; red, more negative than −0.06 au.

**Figure 2:**
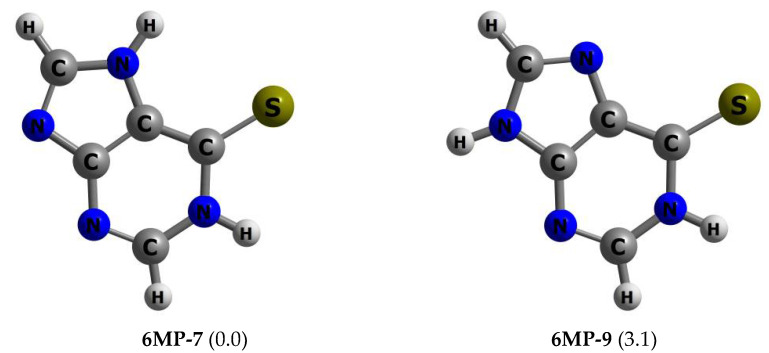
Equilibrium structures of two MP lowest-energy tautomers. Values given in parentheses are their relative energies in the gas phase (kcal/mol at the PBE/cc-pVTZ level).

Numerous experimental and theoretical studies have been devoted to Au_20_, and the pyramidal structure with a *T*_d_ symmetry (Figure 1) is unambiguously assigned as its most stable form [60,61]. The Au_20_ tetrahedron is also well-known as an outstanding indicator in cluster science for its exceptional stability and peculiar 20-electron superatom shell structure [62,63]. Regarding the molecular structure of a 6MP drug, out of several stable configurations [64,65], two planar tautomers—**6MP-7** and **6MP-9,** presented in Figure 2—tend to dominate its ground state population with a large quantity in both the gas phase and aqueous environments, as reported in recent literature [48,64,66]. In a vacuum, **6MP-7** lies ~3 kcal/mol above **6MP-9**, but this energy difference is significantly reduced to ~1 kcal/mol in a water solution (PBE/cc-pVTZ value). Henceforth, both **6MP-7** and **6MP-9** tautomers will be considered for all following calculations and discussion.

As shown in Figure 2, the 6MP molecule exhibits several positions available for binding to the Au_20_ cluster, i.e., the thione head and the nitrogen atoms unbound to hydrogen. During the interaction, the unoccupied anti-bonding orbitals of 6MP can accept electron density from the Au_20_ HOMO. The drug is also able to donate electrons on its lone pairs (HOMO) back to the metal LUMO. Moreover, the cluster can play the role of a proton acceptor and form nonconventional Au···H–N hydrogen bonds in which a net charge is transferred from the gold lone pair to the σ^*^(NH) orbitals [67]. Such an interaction is expected to be an additional factor determining the stability of the resulting products. NBO charges for the Au_20_ cluster (Figure 1) indicated that the corner Au atoms are positively charged, and thus they are more suitable for a nucleophile attack.

**Figure 3:**
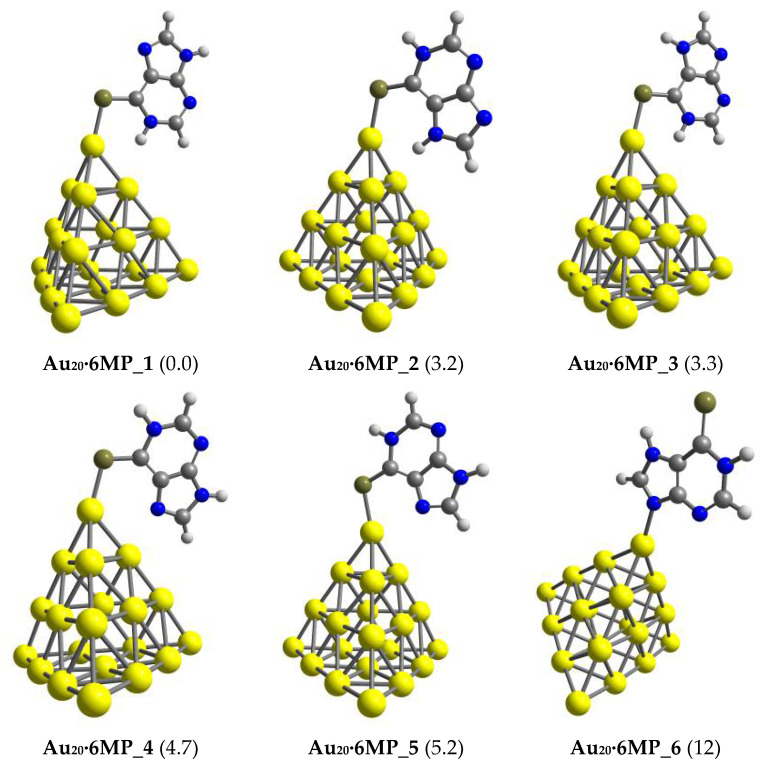
Lower-lying structures of the Au_20_∙6MP complex in the gas phase. Values given in parentheses are their relative energies in kcal/mol from PBE computations. The yellow, gray, blue, lime, and white balls represent Au, C, N, S and H atoms, respectively.

The six stable configurations of the complex formed following binding of 6MP to Au_20_ are presented in Figure 3, while their optimized coordinates are given in Table S1 in the Supplementary Materials. They were denoted as **Au_20_∙6MP_X** with X = 1, 2, 3 … corresponding to an increasing ordering of relative energy (kcal/mol). In general, 6MP prefers to anchor on the vertex of the pyramid, forming a single coordination between the drug and gold cluster (Figure 3). In particular, conformations containing an Au−S bond and a nonconventional hydrogen Au···HN interaction obviously dominate the lowest-lying population of the resulting Au_20_∙6MP complexes. Such structural motifs, i.e., the **Au_20_∙6MP_1**, **Au_20_∙6MP_2,** and **Au_20_∙6MP_3** isomers in Figure 3, strongly compete with each other to be the global minimum structure. In the gas phase, **Au_20_∙6MP_1** is considered the most stable form, lying only about 3 kcal/mol below **Au_20_∙6MP_2** and **Au_20_∙6MP_3** (PBE value). Hence, within the expected error margin of the DFT computations, ± 3 kcal/mol on relative energies, they can coexist in subtle experimental conditions, and the adsorbed molecule is likely to rotate around in axing on the C−S bond.

The next lower-energy isomers **Au_20_∙6MP_4** and **Au_20_∙6MP_5** are formed by anchoring the drug on Au_20_ via the S atom of the pyrimidine ring and the N7 atom of the imidazole moiety. Previously, it was suggested that such interactions are the preferred adsorption modes of 6MP on the gold surface [68]. However, these complexes were computed at the PBE/cc-pVTZ/cc-pVDZ-PP level to be ~5.0 kcal/mol less stable than **Au_20_∙6MP_1** since an N–H⋯Au hydrogen bond was not present, as shown in Figure 3. The remaining structure **Au_20_∙MP_6,** which is constructed by directly binding Au_20_ to a nitrogen atom of the imidazole ring, was computed at 12 kcal/mol less stable. These forms may thus exist only in very small quantities because of their high relative energies. In general, the most favored binding site of 6MP is the S atom, which can be understood on the basis of the hard-soft acid-base (HSAB) theory [69]. Accordingly, the softer S head of 6MP has a higher affinity for soft elements like gold than the harder N head.

For S-bridging complexes, namely **Au_20_∙6MP_1** to **Au_20_∙6MP_5**, the S−Au bond distances varied in the range of 2.41 to 2.44 Å (Table 1), being shorter than the corresponding values of 2.52–2.62 Å predicted for the 6MP adsorption on the Au(001) surface [46]. More specifically, the Au–S bond in the lowest energy structure **Au_20_∙6MP_1** was formed with a distance of 2.41 Å, which was slightly shorter than the sum of covalent radii (2.46 Å) of Au (1.44 Å) and S (1.02 Å) [70]. In addition, the chemical bonds S–C and N–H in **Au_20_∙6MP_1** were lengthened to 1.70 and 1.04 Å, respectively, from the corresponding values of 1.66 and 1.02 Å in free 6MP. For the N-bridging complex, the distance of 2.21 Å for the anchoring bonds Au−N in **Au_20_∙MP_6** was somewhat longer than the sum of the covalent radii of nitrogen (0.75 Å) and gold (1.44 Å).

We evaluated the thermodynamic stability of the Au_20_∙6MP complexes via the binding energy and the changes of Gibbs energy during adsorption. Effects of an aqueous solution on the stability of complexes were also considered using the IEF-PCM model. Table 1 displays the numerical results obtained for the six configurations considered. In the gas phase, the binding energy of **Au_20_∙6MP_1** amounted to 29 kcal/mol and was significantly reduced to 17 kcal/mol for **Au_20_∙6MP_6**. The average $E_{b}$ value of 6MP binding to Au_20_ approximated to ~25 kcal/mol, which was somewhat smaller than the corresponding values obtained for Au_6_∙6MP (28 kcal/mol) and Au_8_∙6MP (32 kcal/mol) complexes [49], but much larger than that of 6MP interacting with a B_24_N_24_ nanocage (16 kcal/mol) [57]. Moreover, because of the entropic effects the Gibbs free energies of the adsorption process became much smaller. The Gibbs free energy of the most stable complex **Au_20_∙6MP_1** amounted to −20 kcal/mol, whereas that of the least stable isomer **Au_20_∙6MP_6** was only ~9 kcal/mol (Table 1).

When the solvent effect was considered, the absolute values of both $E_{b}$ and ΔG turned out to be smaller, indicating that the interactions became weaker and the resulting complexes more breakable. However, the trend was still similar to the gas phase. For example, the Gibbs energy for forming **Au_20_∙6MP_1** in water was ~ −11 kcal/mol as compared to −20 kcal/mol in the gas phase. In addition, the reaction energies to form S-bridging complexes were larger overall than those of the N-bridging counterparts. In particular, the largest change of Gibbs free energy was predicted to be ~ −12 kcal/mol for **Au_20_∙6MP_3**, which was more negative than the value of −3 kcal/mol for the N-bridging complex **Au_20_∙6MP_6**. In general, the 6MP adsorption on the Au**_2_**_0_ surface is likely to be spontaneous, and the products obtained are thermodynamically stable in both the gas phase and aqueous environments, as the computed Gibbs energies remained negative.

### 3.2. The Binding Mechanism and Drug Releasing

For deeper insights into the interaction mechanism, we examined the energies of frontier MOs in the 6MP, Au_20_, and resulting Au_20_∙6MP complexes. The change in the HOMO-LUMO energy gap (${\Delta E}_{g}$) was then calculated as follows (Equation (5)):

(5) $${\Delta E}_{g}=\frac{E_{g_{2}}-E_{g_{1}}}{E_{g_{1}}}\times 100\%$$

where $E_{g_{i}}$ is the energy gap $E_{g}$ of Au_20_ in both the free cluster and the Au_20_∙6MP complexes.

The change of $E_{g}$ is a proper pointer for identifying the presence and attachment of the drug to a metal surface [71]. In fact, the electrical conductivity (σ) of the material is exponentially proportional to a decrease of $E_{g}$ as described by the following Equation (6) [72]:

(6) $$\sigma ={\mathrm{AT}}^{3/2}e^{-E_{g}/2k_{B}T},$$

where *k*_B_ and T are the Boltzmann’s constant and the thermodynamic temperature, respectively, and A is a constant. From a 3-parameter Arrhenius-type Equation (6), a small adjustment of Eg due to the adsorption will result in an enormous change of electric conductivity of the system.

Results summarized in Table 2 clearly pointed out that interaction with 6MP tended to alter the electronic properties of the cluster. At the PBE/cc-pvDZ-PP level, the E_g_ value of Au_20_ was computed to be ~ −5.7 and −5.1 eV in vacuum and aqueous environments, respectively. During the 6MP adsorption process, such a gap was largely declined up to 31% in the gas phase and 20% when the effect of an aqueous solvent was considered (Table 2). According to Equation (6), the electrical conductivity of Au_20_ will exponentially increase with respect to the drop in E_g_ that can be converted to an electrical noise, helping to recognize the drug presence.

The work function Φ, which denotes the energy gap between the Fermi level energy and the void, also plays a crucial role in field emission applications [73]. As a direct result of 6MP attachment, the Fermi level energy decreased from −4.8 eV in the bare Au_20_ cluster to −4.4 eV in the most stable **Au20∙MP_1** complex. Accordingly, the Φ value decreased by nearly 9% (Table 2). This suggested an upgrade in the field emission of the 6MP adsorption and indicated that the gold nanocluster can be used as an Φ–type sensor for detection of 6MP molecules.

**Figure 4:**
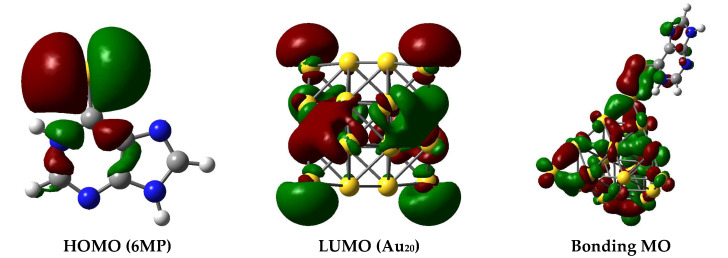
Shapes of frontier orbitals of the Au_20_ cluster and 6MP molecule before and after interaction (Isovalue for the orbital rendering is 0.02 au).

Energies of frontier MOs and their energy difference, i.e., HOMO–LUMO gap (E_g_), are widely used as indicators for analysis of bonding characteristics between the drug and cluster. As mentioned above, the interactions can be characterized by both forward and backward donation bonds. Depending on the symmetry and energy difference of frontier MOs, the former or the latter distribution will be more predominant. As given in Table 2, the energy gap between LUMO of the **6MP-9** tautomer and HOMO of Au_20_ cluster was ~3.5 eV in the gas phase. In contrast, the gap between LUMO of Au_20_ and HOMO of 6MP-9 was significantly reduced to ~1.0 eV. Therefore, the forward donation (6MP→Au_N_) is likely prevailing, and such an interaction is principally characterized by a charge flow from the drug to the Au atoms. While Au atoms acting as electron acceptors gain more negative charges, the 6MP moiety becomes more positively charged. For example, the net charge of Au_20_ in Au_20_-PPX_1 complex was computed to be around −0.4 au (Table S2 of the Supplementary Materials). The nature of Au_20_−6MP bonding was principally similar to the interaction of lone-pair ligands with gold clusters [74], which were mainly formed via the interaction of the unshared electrons in the HOMO of the 6MP molecule with the LUMO of gold metals. The bonding orbital constructed of an overlap between the HOMO of 6MP and the LUMO of Au_20_ is illustrated in Figure 4.

We then examined the ability to detach the drug from the gold cluster in target cells, which is considered as the decisive step in a drug delivery process [49,75]. In a human body, the drug can be segregated from the carrier by either external stimuli or internal stimuli operated within a biologically controlled manner such as the pH and glutathione [76]. Because of an exhaustive lactic production, tumor cells are often more acidic than the normal ones. Indeed, the pH of cancer cells is typically below 6 as compared to values above 7 in blood [77]. In an acidic environment, protons can attack any rich-electron site of the 6MP molecule, but the interaction with the S atom may affect the drug release more significantly. Hence, we inspected the effects of protons on the stability of Au_20_∙6MP complexes by protonation of the thione group, and then carried out geometry optimizations for the resulting Au_20_∙6MPH^+^ products.

**Figure 5:**
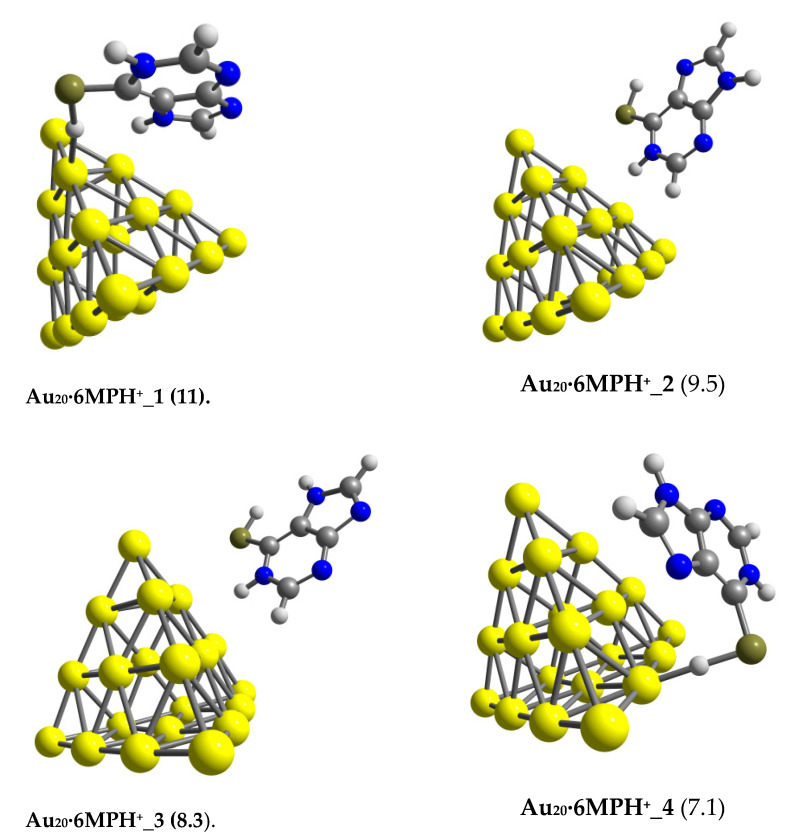
Lower-lying structures of the Au_20_∙6MP complexes in an acidic environment. Values in parentheses are binding energies (kcal/mol, PBE/cc-pVTZ/pVDZ-PP + ZPE). The yellow, gray, blue, lime, and white balls represent Au, C, N, S and H atoms, respectively.

In an acidic environment, the drug binding to gold metals becomes much more breakable. The interaction is now characterized by a weaker hydrogen bond Au⋅⋅⋅H−X (X = S, N), as demonstrated in Figure 5, instead of a covalent Au−S bond as in Au_20_∙6MP. Correspondingly, the binding energy of 6MPH^+^ ions on the Au_20_ surface was substantially reduced to 11 kcal/mol, which was much smaller than the corresponding value of 22 kcal/mol in the neutral solution. This obviously indicated that under acidic conditions, the 6MP binding to a gold surface was getting weaker, resulting in a faster release.

Another important force contributing to a drug release is an inner inducement related to amino acids in the protein matrices [76]. Thiol-containing amino acids such as cysteine and methionine are usually the most favored binding substrates for noble metals [78,79]. Cysteine is a rather strong acid with dissociation constants of p*K*_1_ = 1.7 and p*K*_2_ = 8.3 [80]. Thus, in biological systems, the molecule mostly emerges as the deprotonated forms by proton cleavage of either the thiol or carboxyl group [49]. For deeper insights into the drug release mechanism from gold nanoclusters, we examined the following ligand interchange reaction:

Au_20_∙6MP_(aq)_ + CYSa_(aq)_ → Au_20_∙CYSa _(aq)_ + 6MP_(aq)_.

**Figure 6:**
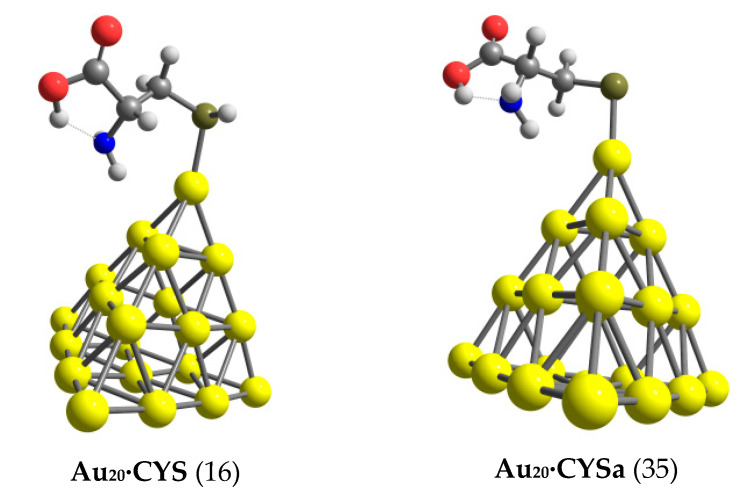
Equilibrium structures for complexes of Au_20_ with cysteine (CYS) and its deprotonated form (CYSa). Values in parentheses are binding energies (kcal/mol, PBE/cc-pVTZ/pVDZ-PP + ZPE). The yellow, gray, blue, lime, red, and white balls represent Au, C, N, S, O and H atoms, respectively.

The most stable forms of Au_20_ with cysteine (CYS) and its deprotonated form, denoted as CYSa, are presented in Figure 6. As recently reported [49,53,75], these species tend to adsorb on the gold surface via the S-atom of the thiol group. In an aqueous solution, the binding energies to Au_20_ were ~35 kcal/mol for CYSa and significantly reduced to ~16 kcal/mol for CYS. These values were much larger than the corresponding values obtained for 6MP adsorption. In fact, the largest binding energy of 6MP to Au_20_ was ~22 kcal/mol in neutral condition, and it substantially dropped to ~11 kcal/mol in acidic solution. Thus, the affinity of gold nanoparticles for cysteine residues was much higher than its affinity for the 6MP molecule. Hence, drug release from a gold surface in target cells is likely to occur because of this internal force.

Moreover, the complexes were able to undergo a desorption process when exposed to light. In principle, stronger interactions between the adsorbent and adsorbate make the recovery process tougher. According to the transition state theory, the recovery time (τ) is exponentially proportional to the binding energy $E_{b}$ as expressed in Equation (7) [81]:

(7) $$\tau =\nu^{-1}e^{E_{b}\;/kT}$$

in which k represents the Boltzman’s constant, ν the attempt frequency, and T the temperature of the system. Equation (7) proves that a larger binding energy E_b_ will disturb the recovery of the device. In agreement with the larger E_b_ values (Table 1), the desorption process time of the 6MP molecule from the Au_20_ surface in a vacuum was generally much longer than that in an aqueous solution.

When applying an attempt frequency of $\nu =6.0\times 10^{14}$ Hz (500 nm), the recovery times for **Au_20_∙MP_1**, **Au_20_∙MP_2**, and **Au_20_∙MP_3** were predicted to be in the range of $2.7\times 10^{4}$–$5.7\times 10^{6}$ s, which were significantly reduced to $0.9\times 10^{1}$–$4.2\times 10^{1}$ s in aqueous solution. The recovery time of Au_20_ in the water solution was thus not too long to hinder the desorption process. The cluster also benefited from a rather high sensitivity as its band gap was much modified during the drug binding. Therefore, this nanostructure could be a promising material for designing tiny sensors for 6MP detection.

### 3.3. SERS Spectra of 6MP on Gold Surfaces

For an interpretation of the SERS phenomenon, two operational mechanisms, i.e., the electromagnetic and the chemical enhancements, are frequently taken into consideration [38,82]. Let us briefly recount a few essential characteristics. The former mechanism is controlled by the molecular symmetry and orientation with respect to the metal surface [83]. Certain Raman signals of molecules near the metallic surface become remarkably enhanced as their intensity is proportional to the squared local electromagnetic field intensity. On the contrary, the enhancement does not occur for vibrations parallel to the metal surface [84]. Moreover, the Raman-scattered light results in an extra enhancement when the Raman mode overlaps with a plasmonic resonance [85]. The latter mechanism, in contrast, mostly refers to the chemical interaction and charge transfer between the nanoparticles and adsorbed molecules [86].

Recently, quantum chemical computations have been widely employed for probing the SERS behaviors including the Raman activities and tensor properties that could provide us with valuable information about the interacting centers and the binding mechanism between adsorbed molecules and the metal surface [87]. For example, the Raman enhancements of pyridine on an Ag electrode were measured and then analyzed with the aid of DFT calculations using the tetrahedral Ag_20_ cluster as a model representing the silver nanoparticles [88]. More recently, similar approaches were also applied to probe the SERS phenomenon of the chlorpyrifos, which is a pesticide using the Ag_20_ cluster as a model for the silver surface [89]. Likewise, the tetrahedral gold Au_20_ cluster was also used as a model to validate the SERS spectrum of pramipexole molecules adsorbed on the gold surfaces [75]. Previous reports are also available on the Raman characteristics of 6MP as well as its SERS phenomenon on gold surfaces [68,84,90]. The molecule is well suited for SERS detection, owing to its high affinity for Ag/Au substrates. More importantly, previous studies have revealed for 6MP as well as for other relevant biochemical species that several proposed hypotheses conflict with each other, and numerous interpretative approaches are needed to address the observations.

**Figure 7:**
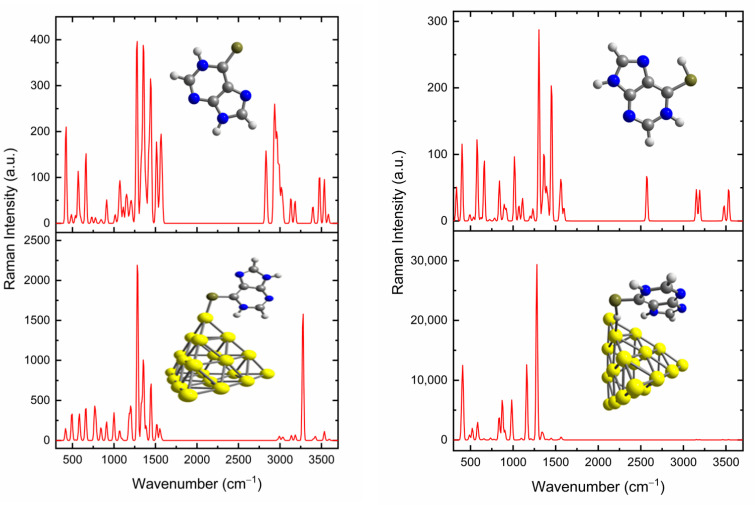
Computed Raman signatures of 6MP (**above**) and its SERS spectra adsorbed on the Au_20_ surface (**below**). Simulations were performed in both neutral (**left**) and acidic (**right**) conditions.

Normal Raman signatures of 6MP simulated in an aqueous solution are presented in Figure 7. As reported experimentally by Kumar et al. [65], several important signals of the free molecule appeared in the region above 3100 and below 1600 cm^−1^. In particular, the N−H stretching mode of the imidazole ring was predicted to vibrate at 3532 cm^−1^, which could be assigned a rather strong peak at 3541 cm^−1^ in the measured spectrum [65]. However, the corresponding mode of the pyrimidine ring, which was located around 3482 cm^−1^, was not observed in experimental data. The C−H stretching mode of the imidazole ring was computed to occur at 3185 cm^−1^, while that of the pyrimidine emerged around 3124 cm^−1^, which was comparable to the measured result of 3104 cm^−1^ [65]. Nonetheless, it was rather difficult to unambiguously assign the intense peaks present in the energy region of 1200–1600 cm^−1^ as they typically resulted from a combination of several vibrations, such as C−X stretching and X−H (X = S, N, C) bending modes. For example, strong signals located around 1360 and 1500 cm^−1^ likely resulted from a coupling of in-plane ring stretching modes with X−H and C−H in-plane bending vibrations. Experimentally, these modes were observed at 1376 and 1540 cm^−1^ [65]. Also in line with experimental observations, some weak bands in the lower energy region were likely to arise from a coupling of ring breathing with the N−H and C−H out-of-plane bending modes.

The simulated Raman signatures for the 6MP thiol formed in acidic solution also agreed well with experimental data acquired in HCl solution [68]. Several strong signals in the fingerprint region peaked at 578, 662, 1017, 1304, 1364 and 1450 cm^−1^, that were comparable to corresponding measured values at 588, 675, 1023, 1330, 1377 and 1449 cm^−1^. In particular, the appearance of the band at 2570 cm^−1^ was due to the thiol (S−H) group [91]. The tetrahedron Au_20_ on the contrary exhibited obvious Raman signals in a narrow frequency range of below 180 cm^−1^ (Figure S1 of the Supplementary Materials), that were much lower in energy than those of the adsorbed molecules.

The normal Raman and SERS spectra of 6MP adsorbed on the Au_20_ cluster surface are compared in Figure 7; those simulations were performed in both neutral and acidic conditions. For the neutral environment, the 6MP species binding to Au metals through the S head exhibited a major contribution to the SERS phenomenon. From the simulated spectra, we saw an extraordinary enhancement of vibrations in the energy region of 1250–1500 cm^−1^. In particular, the strongest enhanced peak at ~1280 cm^−1^ was mostly produced from the N−H bending mode coupled with the C−H deformation and ring breathing. In the experimental SERS spectrum of 6MP on the gold surface recorded in a KCl solution [68], a very strong band at 1257 cm^−1^ was also detected. Another noteworthy aspect was the appearance of a prominent band around 3200 cm^−1^ related to the N−H stretching mode, which was negligible and emerged at higher wavenumbers (3500 cm^−1^) in the normal Raman spectrum of free 6MP.

A comparison of the SERS spectra in both neutral and acidic environments allowed us to explore important deviations in the band intensities and positions owing to the favorable orientation of the adsorbate. The SERS chemical enhancement of 6MP in acidic solution was much stronger than that recorded in the neutral condition. In addition, we found the disappearance of the S–H band near 2600 cm^−1^ on the 6MP SERS spectrum, as such vibration is directly oriented to the gold surface. Experimentally, the SERS spectrum of 6MP in a gold colloidal solution (pH = 4.5) recorded by Pannico et al. [84] showed the strongest peak at 1254 cm^−1^, which was assumed to arise from the ring stretching modes. These authors, moreover, suggested that this enhancement was mainly due to ring-surface π donation and/or surface-ring π* back-donation as a result of a flat orientation on gold surfaces.

In our present calculations, the most enhanced peak was located at 1278 cm^−1^ and the Au⋅⋅⋅H−S coupling turned out to be a predominant factor leading to the SERS enhancement, rather than an aromatic ring–gold surface π overlap. As a result of this nonconventional hydrogen bond, a charge redistribution emerged, giving rise to an SERS chemical enhancement of the thiol form. A further noteworthy feature was found in the high energy region of the spectrum, where the N/C−H stretching vibrations were located. The SERS spectrum, presented in Figure 7, exhibited a complete absence of signals in the 3000–4000 cm^−1^ range, which totally agreed with previous experimental observation [84].

**Figure 8:**
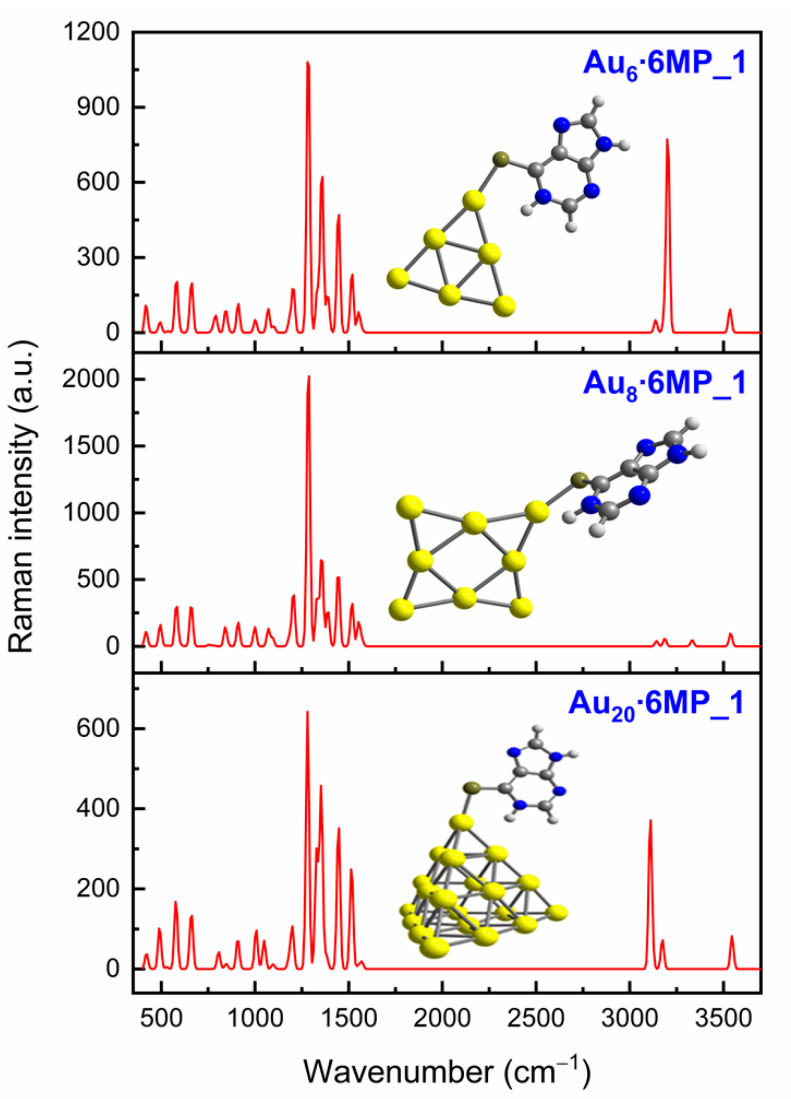
Comparison of 6MP SERS spectra adsorbed on small Au_n_ clusters with n = 6, 8, 20. Simulations are performed in an aqueous solution.

A comparison of SERS spectra adsorbed on some small gold clusters is presented in Figure 8. At first glance, we notice that the spectral positions are almost comparable to each other, while the signal intensity is greatly modified with respect to the cluster size. Small Au_6_ and Au_8_ systems resulted in higher SERS signals than the larger Au_20_ counterpart. In particular, the most enhanced SERS signals of 6MP adsorbed on Au_8_ were primarily related to its lowest HOMO–LUMO energy gap as compared to that of Au_6_ and Au_20_ [60]. To simulate the surface of gold nanoparticles, the use of Au_20_ cluster was expected to provide more reliable results than the smaller sizes Au_6_ and Au_8_ as it had a larger number of gold atoms and a more sphere-like structure. Typically, an icosahedral Au nanoparticle with a diameter of 2–10 nm contains several hundreds to thousands of gold atoms [92]. Overall, in considering their characteristics, these small gold clusters behaved quite well as the simplest models for the gold nanoparticles. The smaller sizes Au_6_ and Au_8_ allowed us to investigate much larger biomolecular targets having well-defined functional groups.

## 4. Concluding Remarks

The adsorption and desorption behaviors along with the SERS chemical enhancement of 6-mercaptopurine (6MP) on a gold nanostructured surface were thoroughly investigated by means of DFT computations. The tetrahedral Au_20_ cluster was used as a simple model to simulate the surface of gold nanoparticles. The effects of an aqueous solution on the structural, energetic, and spectroscopic properties of the resulting complexes were examined with the IEF-PCM model.

In both the gas phase and neutral conditions, the 6MP molecule tended to bind with Au metals via the S-atom of the thione group and was further stabilized by the N−H∙∙∙Au contact. However, in an acidic solution when the drug mainly existed in the thiol form, the interaction was mostly dominated by the weak Au⋅⋅⋅H−S interaction, rather than the stronger covalent Au−S bond. Binding energies were substantially reduced from −29 kcal/mol in a vacuum to −22 and −11 kcal/mol in neutral and acidic solutions, respectively. During the drug adsorption, the energy gap of the adsorbent was greatly modified and likely led to a significant change in the electronic response. Investigation into the frontier MOs also revealed that the forward donation from HOMO(6MP) to LUMO(Au_20_) was a main ingredient of the 6MP−Au chemical bonding.

A comparison of current computed results with previously measured SERS spectra allowed us to verify the preferable orientation of drug molecules on the gold surface. In a neutral solution, both N−H bending and stretching modes were the predominant factors leading to the SERS enhancement as they were directly oriented to the gold surface. In an acidic environment, the most enhanced peak was found in the fingerprint region and was mainly due to the Au⋅⋅⋅H−S bond rather than the aromatic ring-gold surface π overlap as previously predicted. Desorption of the 6MP molecule from a gold surface was triggered by either the low pH in cancerous tissues or the presence of cysteine residues found in protein matrices. Overall, the results demonstrated the high potentiality of gold nanostructures as drug carriers and detectors, particularly in physiological media.

From a methodological viewpoint, the use of small gold clusters, Au_n_ with n being either 6, 8 or 20, as simple models for nanoparticle surfaces tended to provide similar results for the main features of the complex characteristics and their spectra, with some small differences in spectral intensities. The good behavior of smaller-sized clusters as a surface model opens an avenue for the more systematic theoretical investigations of larger biomolecular targets.

## Figures and Tables

**Table 1:** Relative energy (RE), binding energy ($E_{b}$ and Gibbs energy ΔG_298_ in kcal/mol for the 6MP adsorption on A_u20_, along with Au−X=S, N) bond lengths in the resulting complexes (PBE/cc-pVTZ/cc-pVDZ-PP + ZPE).

Complex	RE		$E_{b}$		$\Delta G^{298}$		$r_{Au-X}\;(\text{Å})$
	In a Vacuum	In Water	In a Vacuum	In Water	In a Vacuum	In Water	
**Au_20_∙6MP_1**	0.0	0.0	29.4	22.4	−19.6	−10.5	2.41
**Au_20_∙6MP_2**	3.2	1.0	26.2	21.5	−16.9	−7.82	2.41
**Au_20_∙6MP_3**	3.3	1.0	26.2	21.4	−16.6	−11.5	2.42
**Au_20_∙6MP_4**	4.7	9.9	24.7	12.6	−14.7	−1.91	2.41
**Au_20_∙6MP_5**	5.2	1.4	24.2	21.0	−15.3	−9.64	2.44
**Au_20_∙6MP_6**	12	7.8	17.3	14.7	−9.08	−3.32	2.21

**Table 2:** Energies of HOMO and LUMO, Fermi level energy (E_F_), work function (Φ), energy gap ($E_{g}$ and change of energy gap ($\Delta E_{g}$) upon the 6MP adsorption on the A_u20_ surface. Values are given in eV, except for $\Delta E_{g}$ (%).

Species		In a Vacuum							In an Aqueous Solution				
	HOMO	$E_{F}$	LUMO	$E_{g}$	$\Delta E_{g}$	Φ		HOMO	$E_{F}$	LUMO	$E_{g}$	$\Delta E_{g}$	Φ
**6MP-7**	−5.37	−3.96	−2.55	2.81	-	3.96		−5.52	−4.05	−2.58	2.94	-	4.05
**6MP-9**	−4.93	−3.58	−2.23	2.70	-	3.58		−5.39	−3.91	−2.42	2.98	-	3.91
**Au_20_**	−5.73	−4.84	−3.95	1.78	-	4.84		−5.07	−4.13	−3.18	1.90	-	4.13
**Au_20_∙MP_1**	−5.15	−4.42	−3.69	1.47	−17.4	4.42		−4.81	−4.01	−3.20	1.60	−15.8	4.01
**Au_20_∙MP_2**	−5.28	−4.52	−3.76	1.52	−14.6	4.52		−4.84	−4.03	−3.22	1.62	−14.7	4.03
**Au_20_∙MP_3**	−5.25	−4.50	−3.75	1.49	−16.3	4.50		−4.82	−4.02	−3.22	1.60	−15.8	4.02
**Au_20_∙MP_4**	−4.86	−4.30	−3.73	1.13	−36.5	4.30		−4.74	−3.98	−3.22	1.52	−20.0	3.98
**Au_20_∙MP_5**	−4.87	−4.26	−3.65	1.23	−30.9	4.26		−4.77	−3.98	−3.18	1.60	−15.8	3.98
**Au_20_∙MP_6**	−5.10	−4.35	−3.59	1.51	−15.2	4.35		−4.85	−4.01	−3.16	1.68	−11.6	4.01

## Data Availability

Data sharing is not applicable to this article as no new data were created or analyzed in this study.

## Supplementary Materials

The following are available online. Figure S1: Raman signatures of the tetrahedron Au_20_, Table S1: Geometries and Cartesian coordinates of Au_20_⋅6MP complexes, Table S2: Net charges of Au_20_ moiety in Au_20_⋅6MP complexes.

## Sample Availability

Samples of the compounds in this article are not available from the authors.
