# Supplementary material for: Adsorption/Desorption Behaviors and SERS Chemical Enhancement of 6-Mercaptopurine on a Nanostructured Gold Surface: The Au20 Cluster Model
Source: Molecules. 2021 Sep 6;26(17):5422. doi: 10.3390/molecules26175422 (PMC8434346; doi:10.3390/molecules26175422)
Supplement: Supplementary file 1 [file molecules-26-05422-s001.zip › molecules-1356475-supplementary.pdf]

Adsorption/desorption behaviors and SERS chemical enhancement of 6-mercaptopurine on a nanostructured gold surface: The Au_{20} cluster model

Supplementary Information

Nguyen Thi Nhat Hang,[1] Nguyen Thanh Si,[2] Minh Tho Nguyen[3,*]

and Pham Vu Nhat[2,*]

[1] Faculty of Food Science and Technology, Thu Dau Mot University, Thu Dau Mot, Vietnam; hangntn@tdmu.edu.vn (N.T.N.H)

[2] Department of Chemistry, Can Tho University, Can Tho, Viet Nam; sidoublet276@gmail.com (N.T.S); nhat@ctu.edu.vn (P.V.N)

[3] Institute for Computational Science and Technology (ICST), Ho Chi Minh City, Vietnam; tho.nm@icst.org.vn (M.T.N)

Table S1. Geometric shapes and Cartesian coordinates (angstrom) of Au_{20}·6MP complexes.

Geometries	Cartesian coordinates			
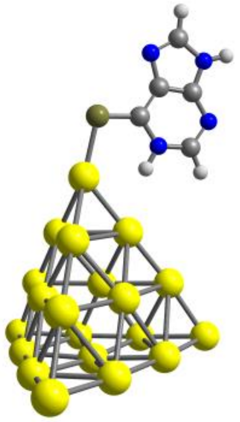 **Au_{20}·6MP_1** (0.0)	79	-2.985591000	-1.620220000	1.347895000
	79	-2.026906000	0.741941000	2.825119000
	79	-1.124942000	2.896065000	1.478715000
	79	-1.124695000	2.896966000	-1.476522000
	79	-2.026618000	0.743789000	-2.824408000
	79	-2.985774000	-1.619269000	-1.348961000
	79	-2.193291000	0.736018000	0.000299000
	79	-0.393685000	-1.765770000	2.825555000
	79	-0.524439000	-1.894658000	-0.000838000
	79	-0.393548000	-1.763498000	-2.826927000
	79	0.507366000	0.595989000	1.595165000
	79	1.453161000	2.750087000	0.001075000
	79	0.508355000	0.597544000	-1.595799000
	79	1.929872000	-1.865138000	-1.422407000
	79	1.929694000	-1.866254000	1.421012000
	79	2.849030000	0.450568000	0.000305000
	79	-2.832977000	-1.539810000	4.064059000
	79	-0.142399000	4.965941000	0.001801000
	79	-2.832933000	-1.536958000	-4.065078000
	79	4.304881000	-1.919763000	-0.000584000
	7	6.463272000	0.508683000	0.000641000
	6	7.290880000	-0.600731000	0.000527000
	6	6.877974000	1.807413000	0.000713000
	16	6.702586000	-2.195610000	0.000503000
	6	8.660844000	-0.236881000	0.000477000
	7	8.134357000	2.189172000	0.000672000
	1	6.078323000	2.549523000	0.000863000
	6	8.981132000	1.137402000	0.000549000
	7	9.800825000	-1.007786000	0.000287000
	7	10.349811000	1.178275000	0.000578000
	6	10.784330000	-0.133149000	0.000336000
	1	11.842112000	-0.381579000	0.000255000
	1	5.442085000	0.316745000	0.000726000
	1	10.917060000	2.020356000	0.000700000
	79	0.876711000	-1.239318000	2.818173000
	79	-1.041439000	-2.967435000	1.346720000
	79	-1.041445000	-2.967443000	-1.346697000
	79	0.876696000	-1.239335000	-2.818168000
	79	2.567752000	0.360349000	-1.447350000
	79	2.567760000	0.360358000	1.447337000
	79	0.919133000	-1.441935000	0.000004000
	79	-2.042038000	-0.614301000	2.821144000

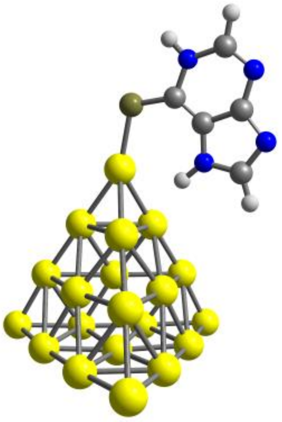 **Au20·6MP_2** (3.2)	79	-0.169898000	1.081039000	1.566740000
	79	1.639903000	2.628706000	-0.000012000
	79	-2.162510000	-0.746416000	0.000007000
	79	-2.042052000	-0.614315000	-2.821130000
	79	-0.169905000	1.081034000	-1.566749000
	79	-0.999313000	3.272631000	-0.000007000
	79	-2.948991000	1.538162000	1.472888000
	79	-2.948998000	1.538155000	-1.472881000
	79	-0.995419000	-2.798426000	4.064927000
	79	-0.995440000	-2.798450000	-4.064906000
	79	4.329343000	1.945365000	-0.000016000
	79	-3.708378000	3.695976000	0.000000000
	16	6.680625000	2.468126000	-0.000027000
	6	7.557632000	1.000129000	-0.000028000
	6	7.139108000	-0.340072000	-0.000027000
	7	8.941218000	1.083655000	-0.000029000
	6	8.075461000	-1.412946000	-0.000027000
	7	5.910646000	-0.944150000	-0.000022000
	6	9.797003000	0.011213000	-0.000028000
	7	9.424284000	-1.238532000	-0.000027000
	7	7.428114000	-2.614696000	-0.000022000
	6	6.141765000	-2.291218000	-0.000031000
	1	4.997543000	-0.465610000	-0.000019000
	1	10.857773000	0.268150000	-0.000029000
	1	5.319598000	-3.001868000	-0.000032000
	1	9.332971000	2.025178000	-0.000029000
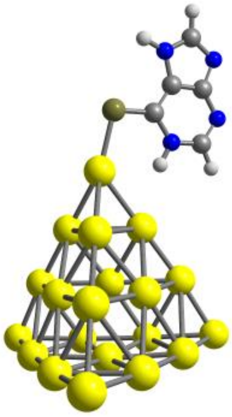 **Au20·6MP_3** (3.3)	79	2.987616000	-1.629704000	-1.335042000
	79	2.035326000	0.728639000	-2.823792000
	79	1.133262000	2.889590000	-1.488245000
	79	1.125181000	2.901822000	1.467648000
	79	2.020111000	0.752059000	2.825778000
	79	2.980962000	-1.618333000	1.361840000
	79	2.192526000	0.732579000	0.001565000
	79	0.398949000	-1.777173000	-2.818220000
	79	0.522611000	-1.895362000	0.008383000
	79	0.384427000	-1.753059000	2.833261000
	79	-0.501671000	0.591262000	-1.598259000
	79	-1.448311000	2.753223000	-0.016326000
	79	-0.511284000	0.605041000	1.589441000
	79	-1.934553000	-1.855514000	1.425419000
	79	-1.927560000	-1.867252000	-1.421520000
	79	-2.850473000	0.458161000	-0.010745000
	79	2.839475000	-1.560339000	-4.052132000
	79	0.149623000	4.966133000	-0.021644000
	79	2.818657000	-1.526344000	4.077469000
	79	-4.300570000	-1.912749000	-0.004235000
	7	-6.470315000	0.560945000	-0.003044000

	6	-7.280845000	-0.552154000	-0.001482000
	6	-6.925369000	1.854270000	-0.003280000
	16	-6.708777000	-2.161314000	-0.001224000
	6	-8.636740000	-0.189630000	-0.000065000
	7	-8.183775000	2.210038000	-0.001973000
	1	-6.139518000	2.611722000	-0.004637000
	6	-9.050667000	1.163516000	-0.000315000
	7	-9.794702000	-0.923050000	0.001796000
	7	-10.419595000	1.244467000	0.001310000
	6	-10.823576000	-0.014154000	0.002565000
	1	-11.862125000	-0.334178000	0.004019000
	1	-5.446571000	0.390669000	-0.004399000
	1	-9.859483000	-1.936712000	0.002484000
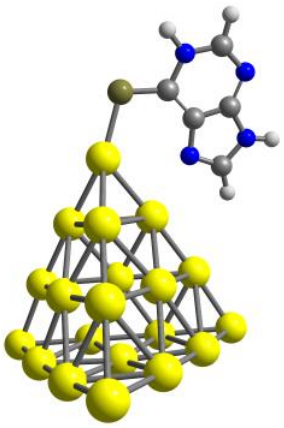	79	2.188838000	0.552991000	-2.703702000
	79	0.778122000	-2.041942000	-2.596452000
	79	-1.669855000	-2.194536000	-1.430970000
	79	-2.945908000	0.248922000	-0.409579000
	79	-1.637571000	2.622314000	-0.492399000
	79	1.050645000	2.775072000	-1.700046000
	79	-0.431634000	0.344807000	-1.675785000
	79	3.193597000	-1.539814000	-0.883967000
	79	2.089854000	0.865055000	0.109971000
	79	0.717181000	3.073150000	1.227735000
	79	0.628942000	-1.916955000	0.225010000
	79	-1.979611000	-1.913896000	1.356599000
**Au_{20}·6MP_4** (4.7)	79	-0.777246000	0.653612000	1.358065000
	79	1.557325000	1.124014000	2.880098000
	79	2.890412000	-1.267334000	1.781703000
	79	0.146325000	-1.470907000	2.979268000
	79	3.334269000	-1.746518000	-3.589412000
	79	-4.165393000	-2.240208000	-0.277883000
	79	-0.182528000	4.930033000	-0.560601000
	79	2.419527000	-0.929851000	4.437683000
	7	-5.564630000	0.817327000	-0.489353000
	6	-6.799705000	0.306542000	-0.154567000
	6	-5.701910000	2.128603000	-0.428394000
	6	-7.288114000	-1.025583000	-0.057123000
	6	-7.698252000	1.363718000	0.113395000
	7	-6.972457000	2.509316000	-0.068701000
	1	-4.902948000	2.839456000	-0.625083000
	16	-6.554644000	-2.528180000	-0.327130000
	7	-8.633845000	-1.040572000	0.317067000
	7	-9.000254000	1.289506000	0.468735000
	1	-7.321573000	3.456132000	0.047843000
	6	-9.416380000	0.050501000	0.555851000
	1	-9.045297000	-1.969396000	0.414450000
	1	-10.449315000	-0.159759000	0.836025000

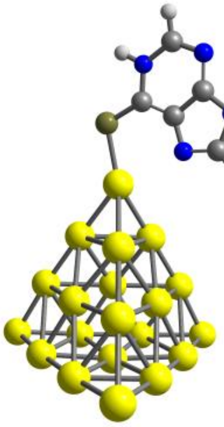 Au$_{20}$·6MP_5 (5.2)	79	0.635955000	-1.421253000	2.823163000
	79	-1.551907000	-2.822647000	1.349911000
	79	-1.551532000	-2.824860000	-1.345577000
	79	0.636849000	-1.425894000	-2.820449000
	79	2.577417000	-0.150953000	-1.425469000
	79	2.576977000	-0.148664000	1.426722000
	79	0.621146000	-1.630464000	0.001508000
	79	-2.133433000	-0.332494000	2.821426000
	79	-0.000502000	1.029803000	1.563911000
	79	2.004345000	2.291337000	-0.001517000
	79	-2.284559000	-0.446534000	0.000097000
	79	-2.132601000	-0.337186000	-2.821349000
	79	0.000006000	1.027322000	-1.565642000
	79	-0.495664000	3.328673000	-0.002767000
	79	-2.677407000	1.935587000	1.472847000
	79	-2.676955000	1.933142000	-1.476709000
	79	-1.474967000	-2.663111000	4.064789000
	79	-1.473761000	-2.669832000	-4.060668000
	79	4.535531000	1.169755000	-0.000314000
	79	-3.100679000	4.183094000	-0.003856000
	16	6.702270000	2.291406000	0.001018000
	6	7.849410000	1.053375000	0.000436000
	6	7.647341000	-0.349172000	-0.000231000
	7	9.207842000	1.365345000	0.000620000
	6	8.769798000	-1.198140000	-0.000582000
	7	6.484979000	-1.075728000	-0.000656000
	6	10.232053000	0.458552000	0.000255000
	7	10.075356000	-0.842389000	-0.000351000
	7	8.251019000	-2.465932000	-0.001203000
	6	6.876155000	-2.333522000	-0.001230000
	1	11.236226000	0.884329000	0.000492000
	1	6.213707000	-3.194434000	-0.001687000
	1	9.436454000	2.359461000	0.001074000
	1	8.787844000	-3.328340000	-0.001594000
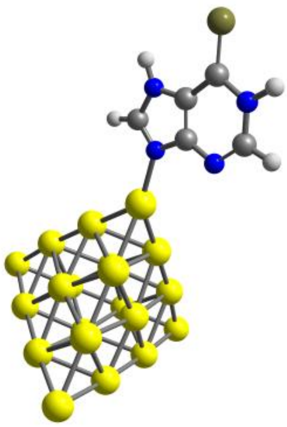 Au$_{20}$·6MP_6 (12)	79	2.324173000	-2.329142000	-1.477747000
	79	-0.081561000	-3.283496000	-0.001706000
	79	-2.326120000	-1.784059000	-0.000725000
	79	-2.439359000	0.713464000	-1.427513000
	79	-0.294995000	1.598374000	-2.821878000
	79	2.225957000	0.003636000	-2.823719000
	79	-0.127892000	-0.929675000	-1.574894000
	79	2.324570000	-2.330784000	1.474965000
	79	2.384418000	0.081919000	-0.000084000
	79	2.121156000	2.555469000	-1.346948000
	79	-0.127623000	-0.931570000	1.574603000
	79	-2.439304000	0.711767000	1.428316000
	79	-0.234567000	1.786014000	0.000987000

	79	2.121339000	2.553912000	1.349570000
	79	2.226132000	0.000626000	2.823478000
	79	-0.295052000	1.595340000	2.823702000
	79	2.306059000	-4.619338000	-0.002617000
	79	-4.603535000	-0.207391000	-0.000130000
	79	2.009651000	2.417005000	-4.063000000
	79	2.009911000	2.412619000	4.065388000
	7	-6.771151000	-0.645001000	-0.000394000
	6	-7.817695000	0.252820000	0.000221000
	6	-7.330861000	-1.855169000	-0.001294000
	6	-9.024802000	-0.453070000	-0.000416000
	7	-7.717841000	1.608620000	0.001261000
	7	-8.687121000	-1.781967000	-0.001363000
	1	-6.782477000	-2.791108000	-0.001943000
	6	-10.291448000	0.186250000	0.000021000
	6	-8.879677000	2.207345000	0.001613000
	1	-9.355234000	-2.549493000	-0.001916000
	16	-11.788050000	-0.535422000	-0.000508000
	7	-10.095354000	1.573621000	0.001012000
	1	-8.923145000	3.297980000	0.002390000
	1	-10.948415000	2.132682000	0.001424000

Table S2. Net charges of Au_{20} moiety in Au_{20}·6MP complexes

Complex	NBO charge (a.u)
**Au_{20}·6MP_1**	-0.35
**Au_{20}·6MP_2**	-0.34
**Au_{20}·6MP_3**	-0.32
**Au_{20}·6MP_4**	-0.38
**Au_{20}·6MP_5**	-0.34
**Au_{20}·6MP_6**	-0.16

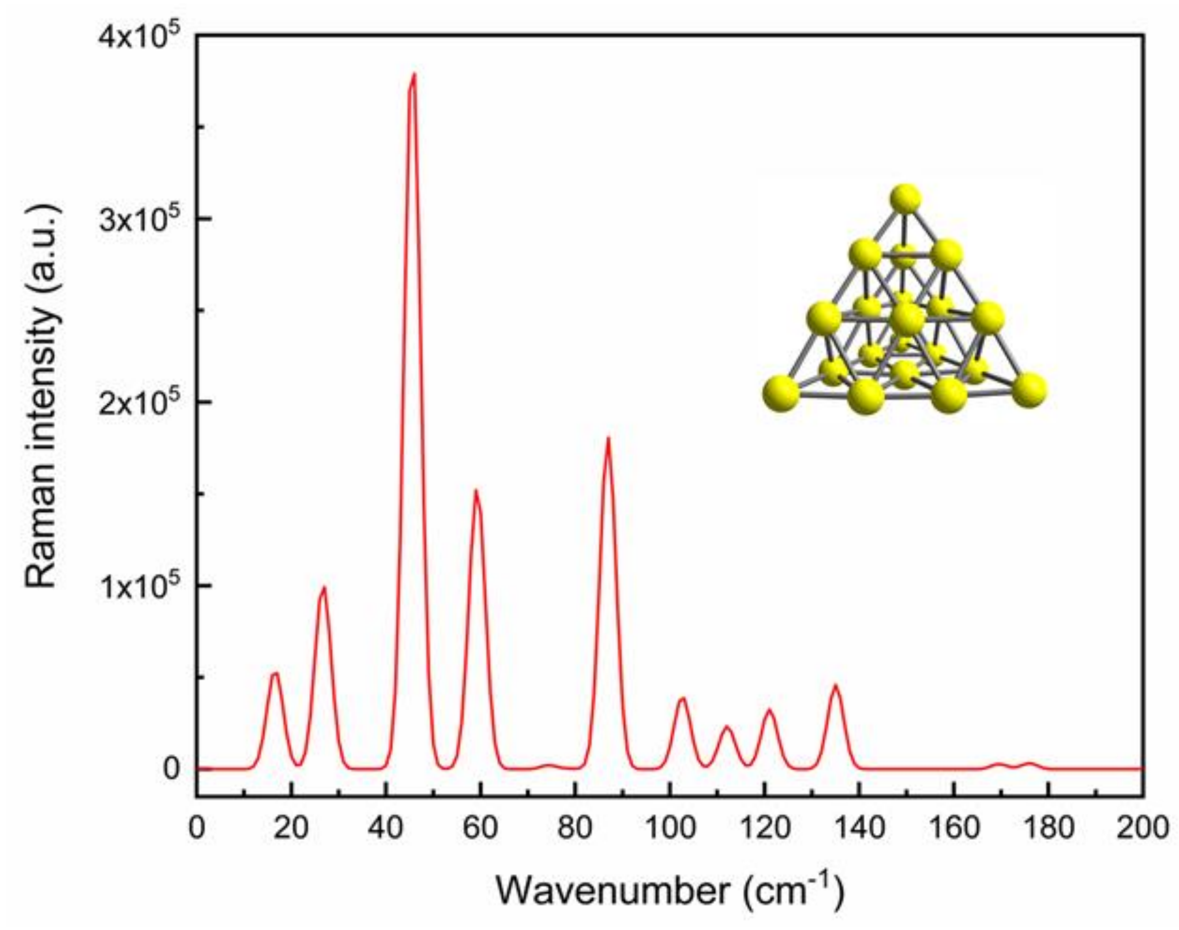

Figure S1. Raman signatures of the tetrahedron Au_{20}
